# We are all ordinary: the shared visual narratives of daily life promote the patients’ positive attitudes toward doctors

**DOI:** 10.1186/s40359-024-01820-8

**Published:** 2024-05-29

**Authors:** Xiaokang Lyu, Shuyuan Zhang, Chunye Fu, Min Yang, Tingting Yang, Fandi Xie

**Affiliations:** 1 Department of Social Psychology, School of Sociology, Nankai University, Tianjin, 300350 China; 2 Computational Social Science Laboratory, Nankai University, Tianjin, China; 3 Shanghai Hopemill Clinic, Shanghai, China; 4 Shanghai Hongkou District Jiangwan Hospital, Shanghai, China

**Keywords:** Doctor-patient relationship, Visual narratives, Identification, Common ingroup identity model

## Abstract

**Background:**

Current research on the doctor-patient relationship primarily focuses on the responsibilities of doctors, with relatively less emphasis on examining the contributions patients can make. As a result, there is an urgent demand for exploring innovative approaches that highlight the active role patients play in cultivating a robust doctor-patient relationship. The purpose of this study was to devise an intervention strategy centered around patients to enhance the doctor-patient relationship. Comics were developed to depict shared narratives encompassing challenging daily life experiences between doctors and ordinary individuals. The study aimed to assess the efficacy of this approach in cultivating positive attitudes toward doctors.

**Method:**

A 3-group design trial was conducted in Shanghai, China. A total of 152 participants were randomly assigned to one of three conditions: the parallel presenting group (*n* = 51), where narratives about a doctor and an ordinary employee were presented side by side in comics; the single presenting group (*n* = 50), where only narratives about a doctor were presented; and the control group (*n* = 51). The outcomes assessed in this study encompassed changes in identification with the doctor portrayed in the comics, perceived intimacy between doctors and patients in reality, and appraisal of the doctor in a prepared doctor-patient interaction situation.

**Results:**

The parallel presenting group exhibited significantly larger increases in identification with the doctor portrayed in the comics, perceived intimacy between doctors and patients in reality, and appraisal of the doctor in a prepared doctor-patient interaction scenario compared to the single presenting group. The observed enhancements in the appraisal of the doctor in a prepared doctor-patient interaction scenario can be attributed to the changes in identification with the doctor portrayed in the comics experienced by the participants.

**Conclusion:**

Our study responds to the doctor-centric focus in existing research by exploring patients’ contributions to the doctor-patient relationship. Using comics to depict shared narratives, the parallel presenting group demonstrated significantly increased identification with the depicted doctor, perceived intimacy, and positive appraisal in prepared scenarios compared to the single presenting group. This underscores the effectiveness of patient-centered interventions in shaping positive attitudes toward doctors, highlighting the pivotal role patients play in fostering a resilient doctor-patient relationship.

**Trial registration:**

Chinese Clinical Trail Registry: ChiCTR2400080999 (registered 20 February 2024; retrospectively registered).

## Background

In recent years, incidents of patient-initiated workplace violence targeting healthcare workers in China have garnered significant public attention [1, 2]. These incidents highlight the underlying tensions between doctors and patients, posing a challenge to the improvement of healthcare system quality. Establishing a harmonious doctor-patient relationship is vital for the effective delivery of healthcare services. Traditionally, the responsibility for improving the doctor-patient relationship has primarily been attributed to doctors, with factors such as apathetic interactions and unprofessional approaches on the part of doctors identified as potential causes of tension [3]. Consequently, there is an increasing emphasis on the implementation of effective doctor-patient communication training [4], the enhancement of humanities education for doctors and medical students [5], and the integration of clinical empathy into clinical skills training courses [6] to address these challenges. Despite these efforts, a crucial aspect often overlooked is the proactive role patients can play in cultivating a robust doctor-patient relationship, prompting the need for innovative approaches that highlight and explore this aspect.

The patient population undeniably assumes a victim identity as they endure the suffering brought about by their illnesses. Conversely, healthcare providers, particularly doctors, also bear their own burdens. They are confronted with substantial responsibilities and face a significant disparity between their heavy workload and relatively low income [7]. Physician burnout is a prevalent systemic issue within the healthcare sector, profoundly impacting professional functioning and individual well-being [8]. Regrettably, physician burnout remains inadequately acknowledged and underreported. It manifests as a state of mental exhaustion, depersonalization, and diminished personal accomplishment, affecting numerous doctors [8–10]. Research suggests that younger physicians, those involved in high-risk procedures, and those experiencing work-life conflicts are particularly susceptible to burnout [11]. Major drivers include excessive administrative tasks, lack of autonomy, and unsustainable workloads [12] Clearly, physician burnout reaches across specialties and career stages, necessitating systemic solutions to this mounting problem.

Physician burnout is often overlooked or misunderstood by patients as simply the complaints of a privileged profession [13]. However, the reality is that both doctors and patients alike suffer under the strains of an overburdened healthcare system [7]. When burnout is perceived as merely a crisis of personal well-being impacting doctors’ work satisfaction, it can elicit limited public sympathy. But in truth, the well-being of physicians and patients is interdependent. By fostering greater empathy and understanding towards doctors, patients can help facilitate more effective doctor-patient communication and mitigate conflicts.

Anderson and colleagues [14] introduced an innovative concept of using comics to juxtapose the everyday experiences of doctors and patients, generating empathy through relatable narratives. For instance, these comics may depict a doctor feeling saddened by lack of time with his daughter, alongside a patient riddled with anxiety before an upcoming exam. This approach expands perspectives beyond the treatment setting, portraying doctors and patients as ordinary individuals balancing their own lives and burdens. Such narratives can provide a foundation for patients to actively contribute to improving doctor-patient bonds.

This concept aligns with the Common Ingroup Identity Model from social psychology. By establishing a common in-group identity between doctors and patients, positive emotions and preferences for one’s own group can extend to the broader shared identity. This shifts the dynamic from an “us versus them” mentality to a more inclusive “we” perspective [15]. Studies have confirmed that common in-group identity can positively predict intergroup help willingness, and is negatively correlated with real threats and negative stereotypes [16], increase the intimacy of group members [17], and shorten social and physical distance [18]. Within the Chinese cultural context emphasizing social harmony, embracing a common doctor-patient identity could prove even more effective in enhancing mutual understanding [19].

Informed by Anderson and colleagues’ [20] idea and the Common Ingroup Identity Model, the present study seeks to experimentally test the potential for visual narratives to improve doctor-patient relationships. We aim to expand the application of visual storytelling to foster a shared identity between doctors and patients beyond the clinical setting. Specifically, this research examines whether juxtaposing the everyday challenges faced by both doctors and ordinary people can enhance readers’ identification with physician characters, perceived intimacy between doctors and patients overall, and appraisals of doctors in simulated interactions. We hypothesize that depicting relatable out-of-office experiences for doctors and patients will establish a sense of common in-group identity, extending positive intergroup attitudes in line with previous findings [16–19]. Additionally, this study explores whether fostering this shared identity through comics can motivate more active, patient-driven efforts to strengthen doctor-patient bonds.

## Method

### Study design

This was a randomized experiment in accordance with the Declaration of Helsinki and received ethical approval from Shanghai Hongkou District Jiangwan Hospital (JW201909) in China.

Many patients’ interactions with doctors are limited to consultation and treatment, which hampers their comprehension of the challenges faced by doctors, such as enduring long-term heavy workloads, navigating tense doctor-patient relationships, coping with prolonged overtime hours, encountering promotion difficulties, and struggling with work-life imbalances. By simultaneously presenting the stressful daily life events of doctors alongside those of ordinary employees, it becomes possible to foster patients’ understanding and identification with doctors, leading to increased tolerance during interactions with doctors. The present study employed comics as an intervention, showcasing the stressful daily life events of a doctor and an ordinary employee side-by-side, with the primary objective of investigating whether this intervention could enhance the doctor-patient relationship within a specific medical interaction setting.

### Participants

The study was conducted at a hospital in Shanghai, with participants comprising inpatients and accompanying family members in the cardiology department. The patient participants were 2-3 individuals per group who had been hospitalized but were well enough to participate. The remaining participants in each experimental group were family members of the hospitalized cardiology patients. These individuals were selected as participants because the state of being in the hospital prompted them to have present personal experience of the doctor-patient relationship issues. This ensured the ecological validity of the study results. Each participant signed a written informed consent form agreeing to participate and confirming that they understood they could withdraw at any time and that their data would remain confidential.

A total of 180 participants were recruited, 28 participants were excluded for failing attention check questions designed to ensure respondents were reading comics carefully. Thus, 152 participants with valid complete data included in the analyses, M_age_ = 35 years (*SD* = 10); age range is 18 to 65 years old; Male = 69, Female = 83. All participants were native Chinese speakers with sufficient language skills and received compensation of 15 yuan (RMB; approximately US $2) for their participation.

### Power and sample size

No previous research was available as a reference for sample size estimation for this study. Therefore, our study was powered to detect medium effect sizes (i.e., Cohen’s *d* = 0.50), with an α error of 5% and a power of 80% with the inclusion of a minimum of 48 participants in each group.

### Intervention

The content of the comics was derived from interviews conducted with ten doctors and ten ordinary employees. During the interviews, participants were specifically instructed to recollect daily work and life events that caused them stress or discomfort. Subsequently, we extracted similar events from the interviews of the ten doctors and ten ordinary employees, which were then adapted into the comics. To ensure the authenticity of the selected events, psychologists, physicians, and other professionals were consulted to evaluate their appropriateness for adaptation. Based on their feedback, ten events were identified and incorporated as the content of the intervention comics.

**Fig. 1:**
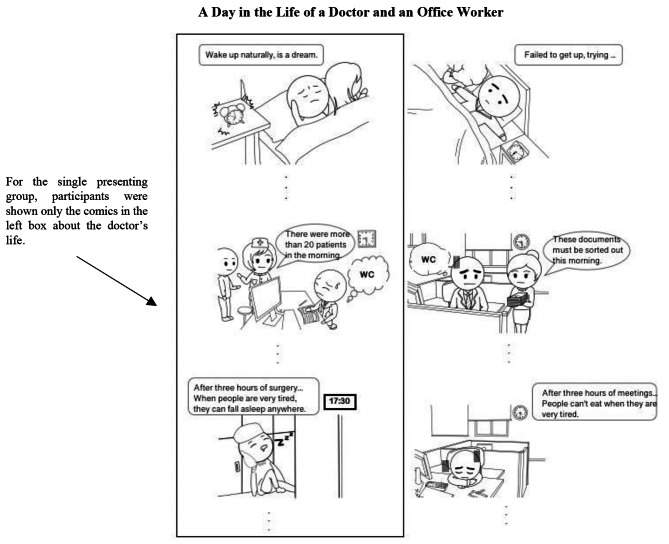
The example comics of the parallel presenting group

The comics were titled “A Day in the Life of a Doctor and an Office Worker” and were presented in a chronological order, depicting ten scenes that spanned from the morning awakening of the doctor or ordinary employee to their evening activities at home, respectively. The narratives of the doctor and the ordinary employee were presented side by side in parallel within the comics. Each set of parallel narratives was paired, such as the doctor facing criticism from a patient and the ordinary employee receiving negative feedback from a client. Participants who read these comics were divided into the parallel presenting group. Additionally, a single presenting group was established as a comparison in this study, where participants read the comic titled “A Day in the Life of a Doctor” featuring only the doctor’s portion from the parallel presenting group comic (Fig. 1).

To minimize the influence of extraneous variables, sketching techniques were utilized in this study. Furthermore, we hired cartoonists to produce the comics to ensure compliance with copyright regulations and avoid potential disputes.

### Measurements

#### Appraisal of the doctors in reality

Participants’ appraisal of doctors in real-life were assessed using a stereotype scale revised by Chinese researchers [20]. This measurement tool was based on the Stereotype Content Model [21], which evaluates individuals’ stereotypes of a particular group in terms of competence (e.g., competent), warmth (e.g., friendly), and ethics (e.g., honest). The scale comprised six adjectives and employed a 5-point Likert scale for scoring. A higher score indicated a more positive perception of doctors. In the present study, the internal consistency of the scale, as measured by Cronbach’s alpha, was 0.92.

#### Perceived intimacy between the doctors and the patients in reality

**Fig. 2:**
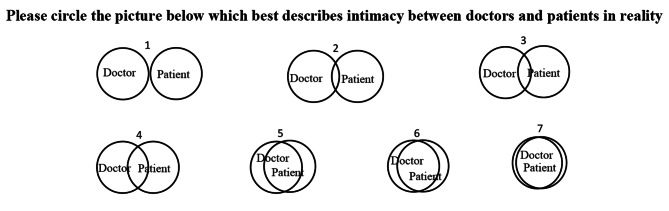
The modified version of the Inclusion of Other in the Self Scale

The Inclusion of Other in the Self Scale [22] was modified to assess the perceived intimacy between the doctors and the patients in reality. The scale is a single-item, pictorial measure of closeness, as the overlap of the circles, so does the closeness of the relation. Seven degree of overlap circles linearly were used for the 7-step, with higher scores indicating that participents perceived higher intimacy between the doctors and the patients in reality (Fig. 2). In the present study, this measurement was used twice for both intervention groups, once before and once after the intervention was delivered.

#### Identification with the doctor in the comics

Identification with the character of the doctor in the comic was assessed using an adapted version of the rating scale employed by de Graaf and colleagues [23]in their research. The measurement consisted of eight items rated on a 7-point scale. Three items pertained to participants imagining themselves in the position of the doctor, for example, “During reading, I imagined what it would be like to be in the position of the doctor”. Two items focused on the participants’ experience of empathy towards the doctor, for instance, “I empathized with the doctor”. Additionally, three items gauged the sense of identifying with the character, such as “In my imagination it was as if I was the doctor”. The internal consistency of the scale, as indicated by Cronbach’s alpha, was 0.91 in the present study.

#### Appraisal of the doctor in a prepared doctor-patient interaction situation

The doctor-patient interaction situation utilized in this study was derived from a notable online conflict that occurred in 2015 between a doctor and a patient in China. The incident involved a celebrity who visited the emergency room due to eye discomfort. The doctor diagnosed the condition as keratitis and prescribed medication. However, the celebrity raised objections, claiming that the doctor did not listen attentively and engaged in a heated exchange with the doctor over the latter’s perceived curt and unresponsive demeanor. Subsequently, the celebrity took to social media to accuse the doctor of irresponsibility and questioned their suitability for the “white angel” title.

**Fig. 3:**
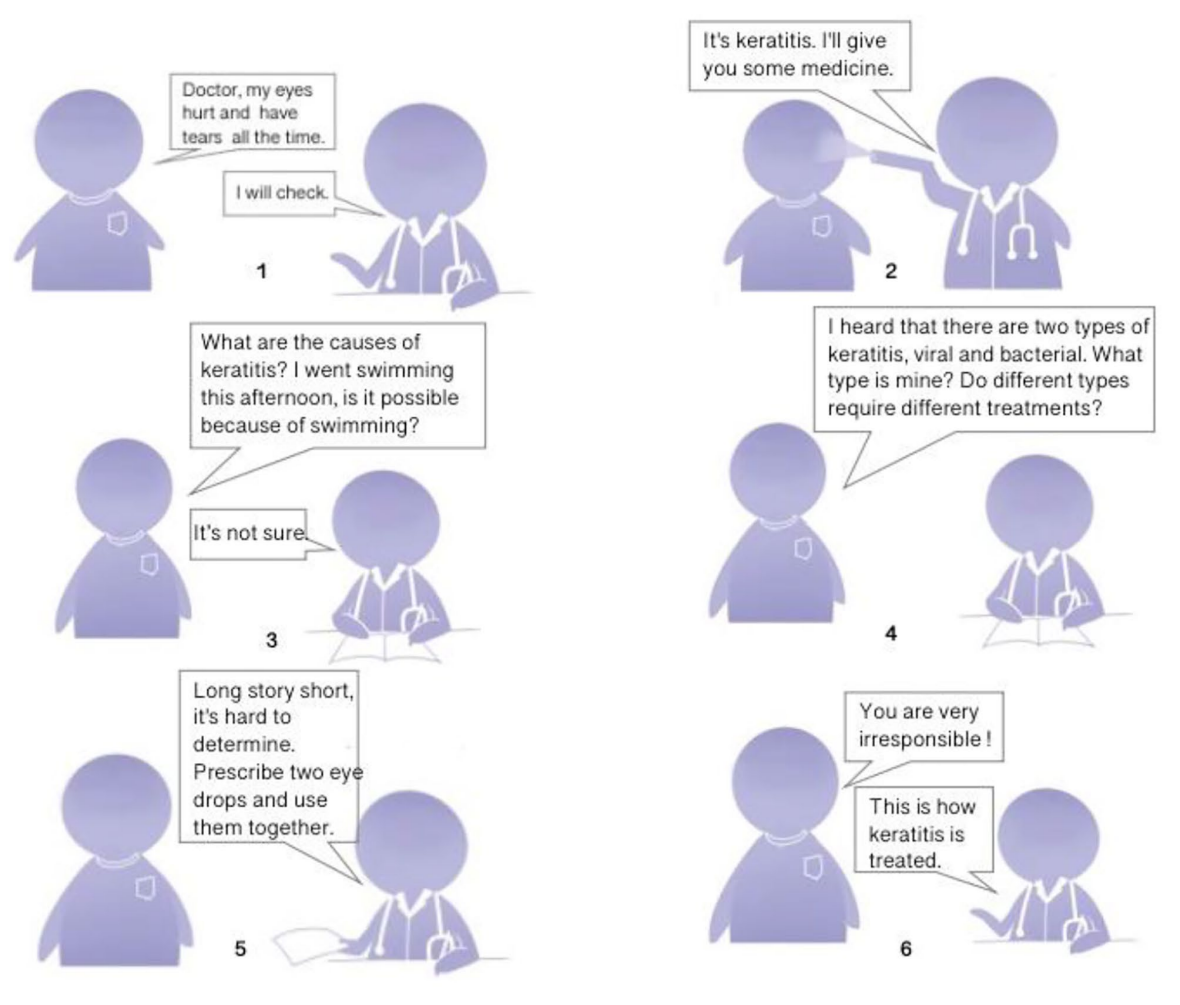
The doctor-patient interaction situation. At ten o’clock in the middle of the night, Mr. Zhang suddenly felt pain in his eyes, and also kept running tears. In an emergency, he had to go to the local general hospital to see the emergency room. The emergency room was crowded, and Mr. Zhang waited in line for a while before his turn came. He had the following conversation with the doctor

The incident generated mixed opinions, with some individuals viewing the celebrity’s actions as overly harsh while others criticized the doctor for being irresponsible. The incident underwent revisions, and experts with medical backgrounds assessed the professionalism of the materials, resulting in the development of an official version of the doctor-patient interaction situation. To ensure clarity and minimize confusion, the scenario materials were presented as a simplified comic strip with a distinct style different from the intervention materials (Fig. 3).

Following the participants’ reading of the materials, they were asked to provide subjective evaluations of the doctor depicted in the materials. The evaluation employed the same measurement used to assess participants’ perceptions of real-life doctors, with the difference being that this time the evaluation pertained to the doctor featured in the scenario materials.

### Procedures

**Fig. 4:**
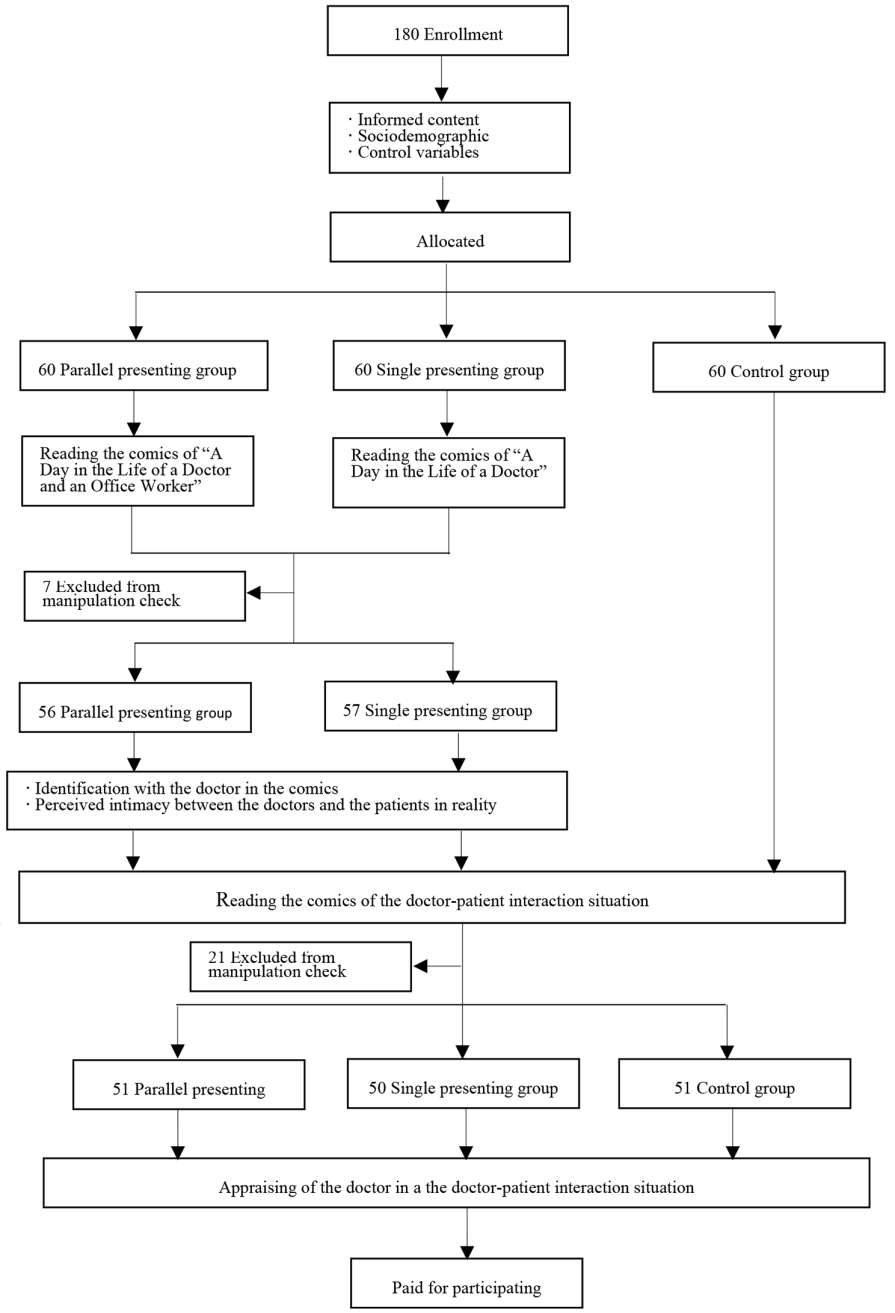
Flow Diagram of Participation and Data Collection

Figure 4 shows the participation flowchart. We conducted our experiment at a hospital in Shanghai, China, with the following steps.

Upon obtaining informed consent, participants were requested to provide sociodemographic information, including gender, age, and education. Baseline questions were then administered, assessing participants’ initial appraisals of doctors and perceived intimacy between doctors and patients in reality. Subsequently, participants were randomly assigned to one of three groups: parallel presenting group, single presenting group, or control group.

Participants in the parallel presenting group and single presenting group were instructed to read the corresponding comics and respond to manipulation check questions. Those who did not pass the manipulation check were excluded from further analysis. Participants in both the parallel presenting group and single presenting group were then asked to answer questions regarding their identification with the doctor depicted in the comics and the perceived intimacy between doctors and patients in reality.

Following this, all participants were provided with a prepared doctor-patient interaction situation to read. After reading, participants were asked to answer manipulation check questions related to the interaction situation, and again, participants who did not pass were excluded. Finally, all participants were asked to appraise the doctor within the interaction situation.

After completion of the experiment, the responses were checked for completeness and paid to the participants.

### Statistical analysis

#### Main outcomes

Descriptive statistics (mean [*SD*] for continuous variables) of participant sociodemographic and control variables, as well as primary outcome measures, were calculated using SPSS, version 22 software (IBM Corporation). Comparisons of outcome measures were performed using independent samples t-test. The effect size, based on the Cohen’s *d* value, was also calculated and interpreted as small (Cohen’s *d* = 0.20–0.49), medium (Cohen’s *d* = 0.50–0.79), or large (Cohen’s *d* ≧ 0.80) [24].

#### Mediation of appraisal of the doctor by identification and perceived intimacy

A mediation analysis was conducted to examine whether identification with the doctor in the comic and perceived intimacy between the doctors and the patients in reality mediated the association between parallel presenting and appraisal of the doctor in the doctor-patient interaction situation, using the PROCESS macro procedure in SPSS, version 22 (IBM Corporation), with 5000 bootstrap samples published by Preacher and Hayes [25]. The unstandardized (B) and standardized (β) regression coefficients are presented for the following equations: (1) regressing the mediators (change in identification and perceived intimacy) on the independent variable (group), (2) regressing the dependent variable (change in appraisal of the doctor) on the independent variable (group), and (3) regressing the dependent variable on both the mediators and the independent variable. Indirect and total effect were also presented, and thus the percentage of the total effect was computed to explain how much of the total effect was explained by the mediation.

## Results

### Sociodemographic and control variables

We analyzed the distribution of the three demographic variables of age, gender, and education, which found that with equal gender and education distribution and with no differences in age among the three groups. When analyzing the appraisal of doctors and perceived doctor-patient intimacy measured pre-test, there was no significant difference between the three groups. So it could be determined that the significance of the subsequent differences came mainly from the experimental intervention and not from the differences between the original groups. Table 1 illustrates the demographic variables for the three groups and provides descriptive results for the pre-test variables.

**Table 1:** Sample characteristics and pre-test variables in each group (*n* = 152)

Variables	Items	parallel	single	control group
		*n* = 51	*n* = 50	*n* = 51
Gender	Female	31 (60.8%)	29 (58.0%)	30 (58.8%)
	Male	20 (39.2%)	21 (42.0%)	31 (41.2%)
Age (years)	Mean (*SD*)	34.1 ± 5.32	37.3 ± 11.57	34.7 ± 10.59
Education	Middle school graduate and below	0	1(2.0%)	2 (3.9%)
	High school graduate	3 (5.9%)	8 (16.0%)	3 (5.9%)
	College degree holder	37 (72.5%)	28 (56.0%)	34 (66.7%)
	Graduate degree holder and above	11 (21.6%)	13 (26.0%)	12 (23.5%)
Perceived intimacy presenting group	Mean (*SD*)	4.10 ± 1.45	3.90 ± 1.28	4.08 ± 1.37
Appraisal of doctors	Mean (*SD*)	3.83 ± 0.72	3.66 ± 0.96	3.82 ± 0.65

### Primary outcomes

**Fig. 5:**
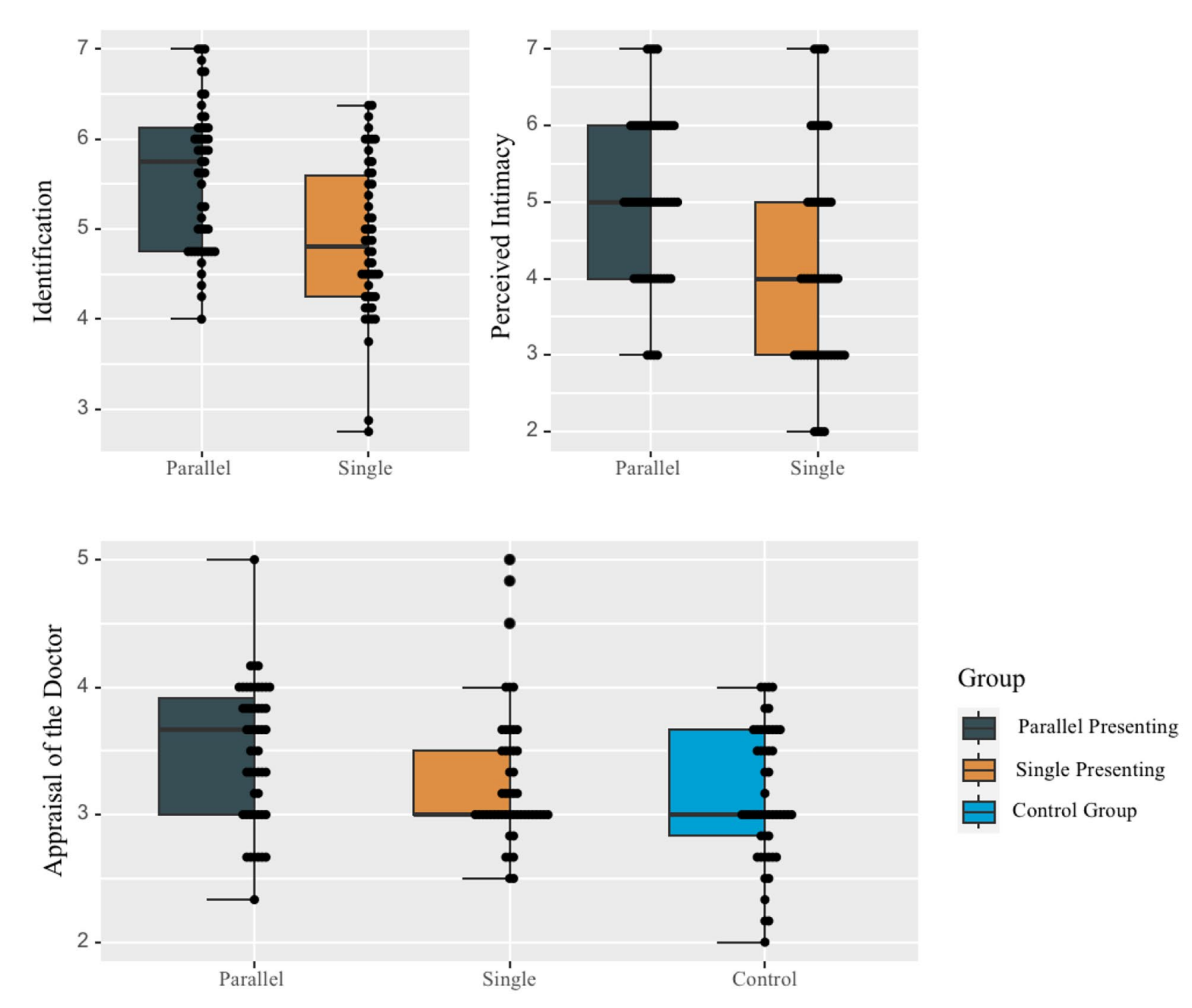
Differences in identification, perceived intimacy, and appraisal of the doctor between or among the groups

Table 2; Fig. 5 shows the descriptive statistics of outcome variables.

**Table 2:** Results of the descriptive statistics of outcome variables

Interventions	Identification	Perceived intimacy	Appraisal of the doctor
	M ± SD	M ± SD	M ± SD
parallel presenting group	5.59 ± 0.80	5.04 ± 1.08	3.50 ± 0.54
single presenting group	4.88 ± 0.85	4.16 ± 1.42	3.27 ± 0.53
control group	—	—	3.15 ± 0.52

Compared with the participants in the single presenting group, participants in the parallel presenting group: experienced significantly greater identification with the doctor in the comics (*t*(99) = 4.33, *p* < 0.001, Cohen’s *d* = 0.86); perceived intimacy between the doctors and the patients in reality higher (*t*(99) = 3.51, *p* = 0.001, Cohen’s *d* = 0.70); had an enhanced appraisal of the doctor in the doctor-patient interaction situation (*t*(99) = 2.20, *p* = 0.03, Cohen’s *d* = 0.37).

For the appraisal of the doctor in the doctor-patient interaction situation, the two intervention groups were compared with the control group, and the results showed that the parallel presenting group appraised the doctors significantly higher (*t*(100) = 3.38, *p* = 0.001, Cohen’s *d* = 0.67); while the single presentation group did not differ from the control group (*t*(99) = 1.15, *p* = 0.25).

### Secondary outcomes

A mediating effect analysis was conducted to determine whether the changes of appraisal of the doctor between parallel presenting group and single presenting group was mediated by identification with the doctor in comics and perceived intimacy between the doctors and the patients in reality. The group (single presenting group = 0, parallel presenting group = 1) was used as the independent variable, identification and perceived intimacy were chain mediators, and appraisal of the doctor was the outcome variable.

**Fig. 6:**
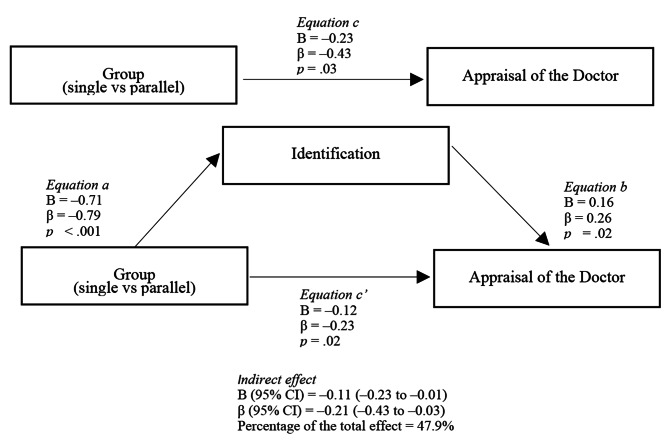
Mediation model of changes in identification mediated changes in appraisal of the doctor

The chain mediating effect of identification and perceived intimacy was not significant (indirect effect: β = 0.001 [95% CI, − 0.05 to 0.06). The analysis yielded a significant model only when identification as mediator (indirect effect: β = −0.21 [95%CI, − 0.43 to − 0.03). Changes in identification explained 47.9% of the improvements in appraisal of the doctor (Fig. 6).

## Discussion

Violence in healthcare settings has become a common occurrence worldwide [26], not only in China. How to establish a harmonious doctor-patient relationship has always been a challenge. By the importance to build trust in doctor-patients relationship as an interdependent phenomenon [27], we believe that solving this dilemma requires efforts not only from physicians but also from patients.

The utilization of comics portraying doctors and patients facing comparable hardships can effectively foster patient identification with healthcare professionals. This identification arises from a mutual recognition of the difficulties confronted by doctors, including long working hours, emotional strain, and the perpetual pursuit of knowledge. When patients perceive doctors as individuals who share their challenges, it establishes a profound connection that promotes empathy and trust. Patients often lack insight into the intricacies and demands of medical practice. By portraying doctors as dedicated, invested, and real individuals, patients develop a deeper understanding of the obstacles physicians encounter. This heightened comprehension can result in increased patience, empathy, and cooperation, ultimately enhancing the general doctor-patient relationship.

We construct the common “ordinary employee” identity of doctors and patients and then explore their influence and mechanism on patient attitudes. This idea is in line with the concept of the Common Ingroup Identity Model [28]. This model suggests that through cooperation, interaction, facing common problems, and emphasizing common destiny, two separate group representations can be transformed into a broader supergroup, i.e., recategorization into a common ingroup identity, which can reduce negative stereotypes and prejudices between the two groups [29]. Similarity increased identification and narrative transportation, which in turn reduced counterarguing, thus resulting in a more positive attitude towards the outgroup [30]. The use of comics to portray doctors as hard workers serve to challenge these stereotypes and reduce stigma. Patients who perceive doctors as fellow hard workers are more likely to view them with compassion and understanding, breaking down barriers that may hinder open communication and trust. Additionally, by highlighting the dedication and effort of doctors, this approach aims to inspire respect and appreciation for the medical profession as a whole.

Our goal is to work on developing interventions that can improve patients’ understanding of doctors in specific medical communication situations, which is not equivalent to a group-level improvement of attitudes toward groups of doctors. Our findings found that parallel presentation improves perceived intimacy at the group level, but that this change does not lead to better evaluations of physicians in specific medical treatments. That is, group-level relationship improvement may have a limited effect on context-specific doctor-patient communication. Further, this study found that identification played a mediating mechanism.

Identification is a vital component of the doctor-patient relationship, enabling public to understand doctors’ perspectives and concerns fully. When patients recognize the dedication and commitment of doctors through the depiction of their hard work, it humanizes healthcare professionals and reduces the perceived power imbalance. This, in turn, establishes a foundation of trust, enhancing communication, shared decision-making, and patient satisfaction. One of the key factors influencing the doctor-patient relationship is the inherent power dynamics within the healthcare system. Patients may feel powerless and intimidated, leading to communication gaps and hindered shared decision-making. By emphasizing the hard work and dedication of both parties, the comics aim to humanize doctors and reduce the perception of an imbalanced power dynamic. This can empower patients to actively engage in their healthcare, fostering a collaborative and equal partnership.

Furthermore, our study is informed by the conceptual framework proposed by Anderson et al. [16], which inspired the creation of a narrative that intertwines the lives of doctors and patients using comics as a medium. By immersing themselves in these cartoons, participants were able to gain a more concrete understanding of the day-to-day experiences of doctors and establish personal connections with their own lives. This experiential understanding is cultivated through a process of active engagement and empathy, enabling participants to “see” and “experience” rather than passively receive information.

It is important to note that graphic medicine represents a distinct genre within the realm of literature, serving as a means to address the communication needs of risk communication and health promotion [31]. Previously, graphic medicine has been utilized as a conduit for patients to express their experiences with specific illnesses, such as dementia [32]. Our research expands the application of graphic medicine by exploring its potential in facilitating improved doctor-patient communication and enhancing the doctor-patient relationship.

The doctor-patient relationship is the cornerstone of effective healthcare delivery, encompassing trust, communication, and collaboration. The doctor-patient relationship has faced several challenges, such as time constraints, communication barriers, and the power dynamics inherent in the healthcare system. Establishing a harmonious connection between doctors and patients has always posed a challenge, requiring mutual understanding and empathy. Moreover, physicians often experience burnout due to the demanding nature of their work, which can negatively impact patient care and the overall doctor-patient relationship. Addressing burnout on an individual level will not be enough in the current healthcare environment [33].

While efforts to improve this relationship have primarily focused on the role of physicians, our findings suggest that patient engagement is equally crucial. By portraying doctors and patients as equally hard-working individuals through the use of comics, this study highlights the potential to enhance patient identification with doctors, foster understanding, and promote recognition.

The study has several strengths, including enhancing doctor-patient relationship through patient engagement. By equating the hard work of both doctors and patients, this approach encourages patients to view healthcare professionals as individuals with their own struggles. Recognizing these challenges, it becomes crucial to explore innovative approaches that can alleviate these issues and promote a more harmonious connection between doctors and patients.

### Limitations

This study, however, has several limitations that should be acknowledged.

Firstly, in order to emphasize the burnout experienced by doctors and evoke empathy, the comic employed focus on negative narrative content. However, it is important to recognize that doctors and ordinary employee also encounter numerous positive events in their daily life. Therefore, further investigation is warranted to explore the impact of narratives with varying emotional value on the improvement of the doctor-patient relationship.

Secondly, although our study was conducted at a hospital site, the materials presented to participants and the examination of their effects on the enhancement of the doctor-patient relationship were carried out in a controlled context. This approach may limit the ecological validity of the findings, as the measurement of behavioral intentions was convenient and did not directly reflect real-life attitudes. Thus, it is recommended to conduct follow-up field experiments with higher ecological validity to gain a deeper understanding of the role of such interventions in real healthcare settings.

Lastly, caution should be exercised when interpreting the results of the mediation analyses, as both the proposed mediator and the outcome were measured concurrently. To establish a more robust causal relationship, future studies should consider employing longitudinal designs or experimental manipulations to examine the mediating role of identification with the doctor over time.

### Implications to research, policy, and practice

The study’s findings carry profound implications across research, policy, and practical dimensions within the healthcare landscape. Research-wise, the demonstrated efficacy of patient-centered interventions, exemplified by the use of comics to portray shared narratives, underscores the imperative of exploring innovative methodologies that recognize and amplify the pivotal role patients play in shaping positive attitudes toward healthcare professionals. This underscores the need for continued exploration and refinement of patient-centric approaches in healthcare studies.

From a policy perspective, the study advocates for a more comprehensive and inclusive outlook in initiatives targeting the enhancement of the doctor-patient relationship. Policies should reflect an understanding of the intricate dynamics of healthcare interactions and actively involve patients in the collaborative development of strategies fostering a harmonious relationship. This suggests a paradigm shift towards policies that engage stakeholders in a more collaborative and patient-inclusive manner.

Practically, healthcare practitioners and educators stand to benefit by incorporating patient-centered communication strategies, as elucidated in this study, into training programs and clinical practices. This integration holds promise for elevating the overall quality of healthcare delivery and enhancing the patient experience. As a result, this study provides actionable insights for practitioners and educators seeking to implement effective and patient-focused communication strategies.

Furthermore, the study’s implications extend beyond the immediate doctor-patient relationship. Amid a burgeoning focus on improving the quality of life for healthcare professionals, the insights gleaned from this research contribute not only to fostering understanding between doctors and patients but also offer patients a valuable perspective on the daily challenges and pressures faced by healthcare providers. This heightened understanding has the potential to alleviate professional stress on doctors, thereby contributing to the creation of a more supportive and empathetic healthcare environment. In essence, the study not only directly benefits the doctor-patient relationship but also indirectly contributes to broader initiatives aimed at enhancing the overall well-being of healthcare professionals, aligning with the evolving landscape of healthcare priorities [34].

## Conclusions

A parallel presenting of intervention the daily narratives of doctors and ordinary employees through comics can be effective in improvement of doctor-patient relationship. These results have relevance and implications for enriching patient-based intervention strategies to improve the doctor-patient relationship.

## Data Availability

Data will be made available on request.

## References

[1] Cai R, Tang J, Deng C, Lv G-F, Xu X-H, Pan J (2019). Violence against health care workers in China, 2013–2016: evidence from the national judgment documents. Hum Resour Health.

[2] Xiao Y, Chen T-T, Zhu S-Y, Zong L, Du N, Jia J (2022). Workplace violence against Chinese health professionals 2013–2021: a study of national criminal judgment documents. Front Public Health.

[3] Venkatesan S, Saji S (2019). Un)bridgeable chasms? Doctor-patient interactions in select graphic medical narratives. J Med Humanit.

[4] Jiang Y, Shi L, Cao J, Zhu L-M, Sha Y, Wei J (2020). Effectiveness of clinical scenario dramas to teach doctor-patient relationship and communication skills. BMC Med Educ.

[5] Fan W, Song Z-Z, Zhang W, Xiao Y-W (2017). Medical humanities play an important role in improving the doctor-patient relationship. BioSci Trends.

[6] Vinson AH, Underman K (2020). Clinical empathy as emotional labor in medical. Soc Sci Med.

[7] Wang S-J, Zhang X-J (2016). Both doctors and patients are victims in China. Int J Cardiol.

[8] Usas H, Weilenmann S, Princip M, Fuchs WJ, van Nuffel M, Spiller TR (2023). Physician-specific symptoms of burnout compared to a non-physicians group. Int J Environ Res Public Health.

[9] West CP, Dyrbye LN, Shanafelt TD (2018). Physician burnout: contributors, consequences and solutions. J Intern Med.

[10] Youssef D, Youssef J, Abou-Abbas L, Kawtharani M, Hassan H (2022). Prevalence and correlates of burnout among physicians in a developing country facing multi-layered crises: a cross-sectional study. Sci Rep.

[11] Brian EL, Johanna LC (2018). Physician burnout: the hidden health care crisis, clinical gastroenterology and hepatology. Clin Gastroenterol Hepatol.

[12] Dewa CS, Loong D, Bonato S, Trojanowski L (2017). The relationship between physician burnout and quality of healthcare in terms of safety and acceptability: a systematic review. BMJ Open.

[13] Epstein RM, Privitera MR (2016). Doing something about physician burnout. Lancet.

[14] Anderso PF, Wescom E, Carlos RC (2016). Difficult doctors, difficult patients: building empathy. J Am Coll Radiol.

[15] Dovidio JF, Gaertner SL, Saguy T (2009). Commonality and the complexity of we: social attitudes and social change. Personality Social Psychol Rev.

[16] Zhou T-S, Hu Q, Cui L-J (2018). Common ingroup identity and intergroup helping:the mediating effect of intergroup threat. Psychol Res.

[17] Moss SM (2017). Identity hierarchy within the Sudanese superordinate identity: political leadership promoting and demoting subordinate groups. Political Psychol.

[18] Alnabulsi H, Drury J (2014). Social identification moderates the effect of crowd density on safety at the Hajj. Proc Natl Acad Sci USA.

[19] Deng X, Long S-Y, Sheng Y-L, Zhao H-H (2023). He w.Influence and mechanisms of common ingroup identity on competitive victimhood in doctor-patient relationships. Acta Physiol Sinica.

[20] Guan J, Cheng J-T (2011). Dimensionality and measure of stereotype content model and influence of involvement. Chin J Clin Psychol.

[21] Fiske ST, Xu J, Cuddy AJC, Glick P (1999). Dis)respecting versusn (dis)liking: status and interdependence predict ambivalent stereotypes of competence and warmth. J Soc Issues.

[22] Aron A, Aron EN, Smollan D (1992). Inclusion of other in the self scale and the structure of interpersonal closeness. J Pers Soc Psychol.

[23] de Graaf AD, Hoeken H, Sanders J, Beentjes JWJ (2012). Identification as a mechanism of narrative persuasion. Commun Res.

[24] Cohen J (1992). A power primer. Psychol Bull.

[25] Preacher KJ, Hayes AF (2004). SPSS and SAS procedures for estimating indirect effects in simple mediation models. Behav Res Methods Instrum Comput.

[26] Jesús C, Cruz SPDL (2020). A worldwide bibliometric analysis of published literature on workplace violence in healthcare personnel. PLoS ONE.

[27] Petrocchi S, Iannello P, Lecciso F, Levante A, Antonietti A, Schulz PJ (2019). Interpersonal trust in doctor-patient relation: evidence from dyadic analysis and association with quality of dyadic communication. Soc Sci Med.

[28] Gaertner SL, Dovidio JF, Anastasio PA, Bachman BA, Rust MC (1993). The common ingroup identity model: recategorization and the reduction of intergroup bias. Eur Rev Soc Psychol.

[29] Dovidio JF, Gaertner SL, Saguy T (2007). Another view of we: majority and minority group perspectives on a common ingroup identity. Eur Rev Soc Psychol.

[30] Igartua JJ, Cachón-Ramón D (2023). Personal narratives to improve attitudes towards stigmatized immigrants: a parallel-serial mediation model. Group Process Intergroup Relat.

[31] Green MJ (2022). Graphic medicine-the best of 2022. JAMA.

[32] Kovan SB, Soled DR (2023). A disembodied dementia: graphic medicine and illness narratives. J Med Humanit.

[33] Privitera MR, Rosentein AH, Plessow F, LoCastro TM (2014). Physician burnout and occupational stress: an inconvenient truth with unintended consequences. J Hosp Admin.

[34] Krishnan A, Odejimi O, Bertram I, Chukowry PS, Tadros G (2022). A systematic review of interventions aiming to improve newly-qualified doctors’ wellbeing in the United Kingdom. BMC Psychol.

